# Accessible Region Conformation Capture (ARC-C) gives high-resolution insights into genome architecture and regulation

**DOI:** 10.1101/gr.275669.121

**Published:** 2022-02

**Authors:** Ni Huang, Wei Qiang Seow, Alex Appert, Yan Dong, Przemyslaw Stempor, Julie Ahringer

**Affiliations:** 1 The Gurdon Institute and Department of Genetics, University of Cambridge, Cambridge CB2 1QN, United Kingdom

## Abstract

Nuclear organization and chromatin interactions are important for genome function, yet determining chromatin connections at high resolution remains a major challenge. To address this, we developed Accessible Region Conformation Capture (ARC-C), which profiles interactions between regulatory elements genome-wide without a capture step. Applied to *Caenorhabditis elegans*, ARC-C identifies approximately 15,000 significant interactions between regulatory elements at 500-bp resolution. Of 105 TFs or chromatin regulators tested, we find that the binding sites of 60 are enriched for interacting with each other, making them candidates for mediating interactions. These include cohesin and condensin II. Applying ARC-C to a mutant of transcription factor BLMP-1 detected changes in interactions between its targets. ARC-C simultaneously profiles domain-level architecture, and we observe that *C. elegans* chromatin domains defined by either active or repressive modifications form topologically associating domains (TADs) that interact with A/B (active/inactive) compartment-like structure. Furthermore, we discover that inactive compartment interactions are dependent on H3K9 methylation. ARC-C is a powerful new tool to interrogate genome architecture and regulatory interactions at high resolution.

The development and application of chromosome conformation capture methods have been instrumental in shaping our understanding of genome topology (Dekker et al. 2002; Sati and Cavalli 2017). The basic premise is that if two regions of the genome are in close proximity within the nucleus, they can be ligated together after the DNA is fragmented. The products generated by proximity ligation can then be determined using sequencing or PCR-based methods. A diverse array of “C” methods have been developed, and their use has revealed interactions between regulatory elements, self-interacting topologically associating domains (TADs), and a “compartment” structure of the genome in which regions of similar activity interact like with like (Dekker et al. 2013).

The Hi-C method enables genome-wide assay of chromatin interactions (Lieberman-Aiden et al. 2009). In most Hi-C-type methods, DNA is fragmented relatively uniformly using restriction or other enzymes for capture of interactions between all regions of the genome (Davies et al. 2017). These genome-wide Hi-C methods are powerful for mapping chromatin domain structures such as TADs, but because of low resolution at reasonably achievable sequencing depth, they are not able to profile interactions between individual promoters and enhancers (Davies et al. 2017). These instead are typically mapped by enriching a Hi-C library for regions of interest, such as collections of promoters or regions bound by transcription factors, using oligo-based capture methods, for example, Capture-C (Hughes et al. 2014), Capture Hi-C (Mifsud et al. 2015), or Targeted DNase Hi-C (Ma et al. 2015). This requires the user to choose and synthesize regions to target and limits the assay to specific subsets of elements, adding expense and complexity to the assay.

Chromatin at regulatory elements is known to be relatively accessible to nucleases, which has enabled their mapping using sensitivity to DNase I or Tn5 transposition (Tsompana and Buck 2014). A higher concentration of DNase I has been used to fragment chromatin uniformly in DNase-Hi-C and Targeted DNase Hi-C methods (Ma et al. 2015). We reasoned that using a low concentration of DNase I to bias cutting toward accessible chromatin would enrich a chromatin interaction library for interactions between regulatory elements and enable their profiling while still having sufficient information for interrogating larger-scale architecture. This would avoid limiting high-resolution information to user-defined regions (as in target capture methods) and allow mapping domain structure in the same experiment. Using this principle, we developed Accessible Region Conformation Capture (ARC-C).

## Results

**Figure 1.**
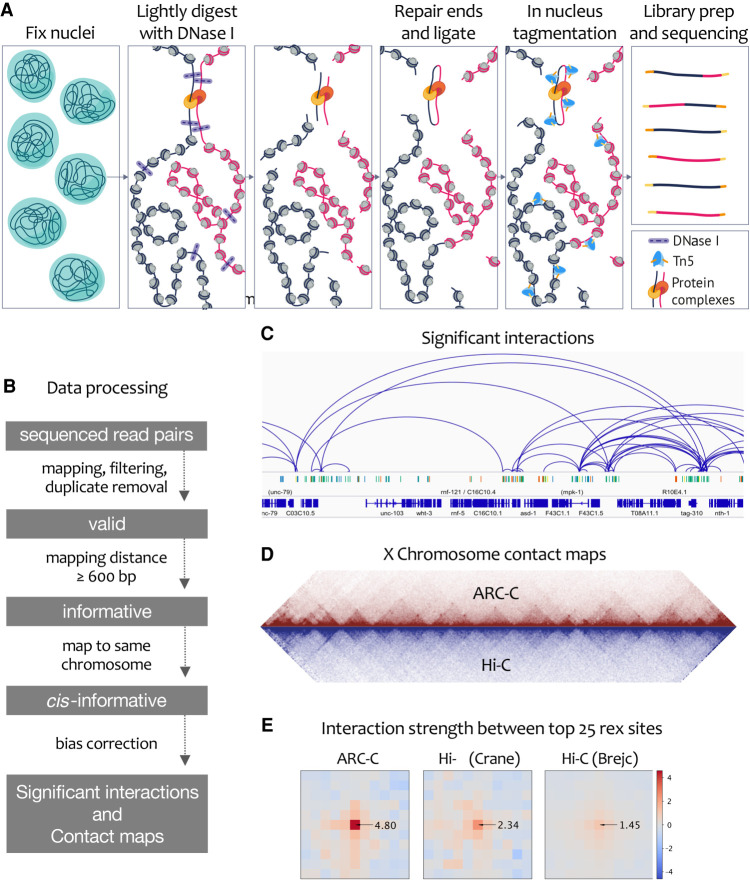
ARC-C method and comparison with Hi-C. (*A*) ARC-C cartoon. (*B*) Data processing steps. (*C*) Integrative Genomics Viewer (IGV) (Robinson et al. 2011) screen shot showing significant interactions in a 275-kb window of Chr III (4,075,000–4,300,822). Regulatory elements (protein-coding promoters, red; unassigned promoters, yellow; enhancers, green; unknown activity, blue) (Jänes et al. 2018) and genes are displayed *below*. (*D*) Comparison of ARC-C and Hi-C X Chromosome contact maps. Hi-C data are from Crane et al. (2015). (*E*) Aggregate contact analysis plots (Rao et al. 2014) showing signal between top 25 rex sites (Crane et al. 2015) at 10-kb resolution and a distance range of 100 kb to 4 Mb for ARC-C (this study) and two *C. elegans* Hi-C maps (Crane et al. 2015; Brejc et al. 2017). Arrows indicate the linear enrichment of rex–rex interactions (at the central 10-kb square) relative to interactions between other regions. rex–rex interaction strength was statistically significant (*P* < 0.001; permutation test, see Methods) in all three maps.

The steps of ARC-C are illustrated in Figure 1A (Methods). Nuclei fixed with formaldehyde are treated with a relatively low concentration of DNase I to bias for cutting at accessible chromatin and give maximal recovery of interactions at regulatory elements (Supplemental Fig. S1; Methods). Ends are then repaired and DNA ligated to join ends in close proximity. The library is then made in nucleus using Tn5 tagmentation (Buenrostro et al. 2013), amplified, size-selected for inserts <400 bp, and paired-end sequenced. For data processing, we first identify “valid” high-quality uniquely mapping read pairs. We then define “informative” read pairs that have captured ligation events as those mapping >600 bp apart or to different chromosomes. Finally, we use “*cis*-informative” read pairs (those mapping to the same chromosome) to construct chromosome-wide contact maps and to call significant interactions following bias correction (Fig. 1B–D; Supplemental Fig. S2; Methods).

We applied ARC-C to *Caenorhabditis elegans* L3 chromatin, preparing libraries from three biological replicates. Data from all replicates were highly concordant (Supplemental Fig. S2). To increase the power to profile interactions, all *cis*-informative reads were pooled, resulting in 12 million read pairs for analysis (Supplemental Fig. S2). As expected, *cis*-informative read pairs were enriched at regulatory elements—43.7% of reads overlap regulatory elements (REs), which comprise 21.1% of the genome—and REs are from Jänes et al. (2018). *Cis*-informative read pairs are produced by ARC-C with an efficiency similar to those of capture methods (Supplemental Fig. S3).

Previous studies of *C. elegans* genome topology using Hi-C identified and characterized large roughly 200-gene self-interacting domains on the X Chromosome regulated by the dosage compensation complex (DCC) (Crane et al. 2015; Brejc et al. 2017). Large domains were also observed on autosomes, although they were weaker, but smaller self-interacting TADs similar to those in *Drosophila* and vertebrate genomes, which typically contain one to several genes, were not detected (Dixon et al. 2012; Nora et al. 2012; Sexton et al. 2012; Dekker and Heard 2015; Brejc et al. 2017). The X Chromosome domain boundaries were shown to be enriched for DCC binding at recruitment element on X (rex) sites, and enrichment for contacts between rex-containing regions was observed (Crane et al. 2015; Brejc et al. 2017).

We found that ARC-C recapitulated the X Chromosome domains and insulation profiles observed by Hi-C and more sensitively detected interactions between rex sites (Fig. 1D,E; Supplemental Figs. S4, S5). ARC-C maps and insulation profiles from the autosomes are also highly similar to Hi-C maps including detection of the previously reported preferential interactions within the large central and two distal chromosome blocks of autosomes (Supplemental Figs. S4, S5; Crane et al. 2015). These results show that ARC-C can profile large-scale domain architecture and suggest that it can improve detection of specific interactions between regulatory elements.

We next investigated the potential formation of smaller TAD-like domains. Although these were not clearly visible in the contact matrix, we considered that genomic domains defined by chromatin modifications might form self-interacting domains, because in other animals, marking patterns within individual TADs and small compartmental domains are relatively uniform (Sexton et al. 2012; Rowley et al. 2017). In *C. elegans*, histone modification domains segment most of the autosomal genome into active and H3K27me3 (aka “regulated”) domains (Gaydos et al. 2012; Evans et al. 2016). Active domains are enriched for broadly and germline active genes marked by H3K36me3 and other modifications associated with gene activity, and the alternating H3K27me3 domains cover genes that have regulated expression (Gaydos et al. 2012; Evans et al. 2016). Both domain types have a median gene number of three, with median lengths of 22 kb for active domains and 19 kb for H3K27me3 domains (Evans et al. 2016). The levels of valid ARC-C signals were only slightly higher in active domains compared to H3K27me3 domains, enabling its use to investigate whether the active and H3K27me3 chromatin domains are spatially separated and form TAD-like structures (Supplemental Fig. S6A,B).

**Figure 2.**
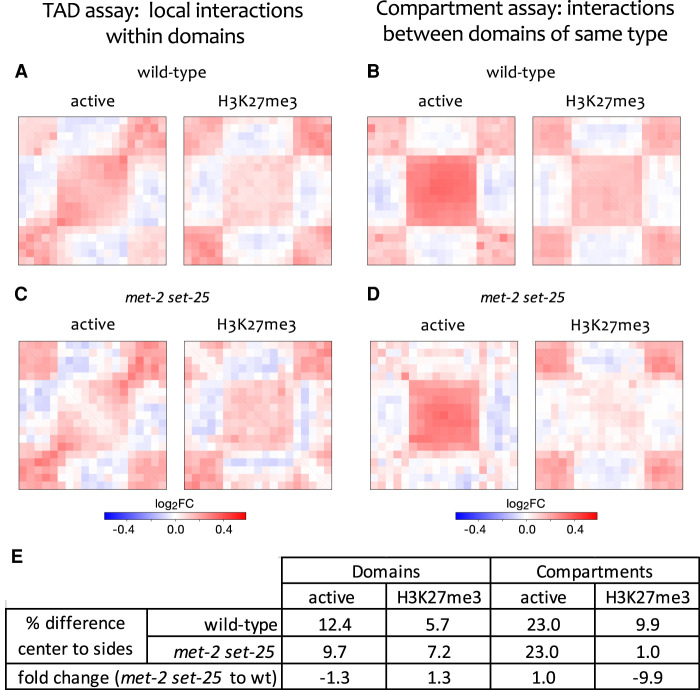
Chromatin modification domains form TADs that have compartment structure. (*A*,*C*) Active and H3K27me3 chromatin modification domains were aligned and contact map signal aggregated in the aligned regions and neighboring regions. Higher signal in the *central* square shows enrichment for within domain interactions, indicative of TAD structure. (*B*,*D*) All possible pairs of inter-domain contacts in the range of 50 kb to 2 M were aligned and signal aggregated as in *A* and *C*. Higher signal in the *central* square shows that domains interact more frequently with domains of the same type than with domains of opposite types, indicative of compartment structure. (*E*) Quantification of enrichment in contact frequencies (as percent differences) for domains and compartments in wild-type and *met-2 set-25* mutants. For domains, the percent difference in contact frequency within domains versus neighboring regions is shown. For compartments, the percent difference in contact frequency between domains of the same type versus domains of different type is shown. See Methods for details.

To investigate whether the active and H3K27me3 chromatin domains are spatially separated and form TAD-like structures, we visualized and compared interaction frequencies in aggregate within active or H3K27me3 domains and their neighboring chromatin. Supporting the spatial separation of both domain types, we found enrichment for interactions within domains, visible as a central square of higher signal (Fig. 2A,E). To further test topological separation, we calculated average insulation scores for active and H3K27me3 domains, where low values indicate regions with low contact frequency (Crane et al. 2015). We observed local minima of insulation profiles flanking active and H3K27me3 domains, supporting the existence of TAD boundaries (Supplemental Fig. S6C). These results indicate that active and H3K27me3 domains have TAD structure, because they are enriched for self-interactions and insulated. The compact nature of the *C. elegans* genome and the relative weakness of these domains compared to TADs in *Drosophila* and mammals may have prevented their detection from de novo analyses.

We next investigated whether domains of the same type (active or H3K27me3) interact with each other to form an A/B (active/inactive) compartment-like structure. For these analyses, we aggregated ARC-C signal either between active domains or between H3K27me3 domains. This revealed that domains show preferential interactions with those of the same type (Fig. 2B,E). Although weaker, domains and compartments detected using ARC-C are also apparent in similar analyses of Hi-C data (Supplemental Fig. S7B). We conclude that *C. elegans* active and H3K27me3 chromatin domains form TADs and that TADs of the same type interact with each other to form a compartment-like structure similar to A/B (active/inactive) compartments of other animals. We note that compartment strength appears to be higher than that of TAD strength. This may be related to the apparent lack of insulator proteins in *C. elegans* (e.g., Heger et al. 2009). A possibility is that compartment interactions between domains of like type contribute to the observed TAD structures.

The mechanisms of compartment formation are not well understood, and few factors that affect compartment structure are known. We considered that histone modifications may be important because they largely differ within active and inactive compartments (Lieberman-Aiden et al. 2009). H3K9 methylation is a good candidate because heterochromatic H3K9 di- and trimethylation is predominantly found in inactive compartments, and interactions within heterochromatin have been suggested to drive compartment structure (Falk et al. 2019).

The two distal regions of *C. elegans* autosomes contain most of the H3K9 methylation and associate with the nuclear lamina, whereas the central region contains little H3K9 methylation (Ikegami et al. 2010; Liu et al. 2011). Active and H3K27me3 domains alternate across both distal and central chromosomal regions, with H3K27me3 marking largely coinciding with H3K9me3 in the distal arm regions (Gerstein et al. 2010; Liu et al. 2011; Evans et al. 2016; Ahringer and Gasser 2018). H3K9me2 and H3K9me1 are also predominantly found in the distal arm regions, but are found in both active and H3K27me3 domains (Gerstein et al. 2010; Liu et al. 2011; Evans et al. 2016; Ahringer and Gasser 2018).

Nearly all H3K9 methylation in *C. elegans* is generated by two enzymes: MET-2 (a histone methyltransferase similar to human SETDB1) and SET-25 (Towbin et al. 2012). Mutants lacking both enzymes are viable and fertile but have undetectable H3K9 methylation and lose nuclear lamina association of chromatin normally marked by H3K9 methylation (Towbin et al. 2012), indicating a potential role of H3K9 methylation in large-scale nuclear organization.

To investigate whether H3K9 methylation plays a role in compartment structure, we performed ARC-C on *met-2 set-25* double mutants. We observed that active and H3K27me3 chromatin domains formed TADs relatively normally, although there were small changes in interaction strength (Fig. 2C,E). Compartment interactions between active domains were also maintained (Fig. 2D,E). In contrast, the *met-2 set-25* double mutants lacking H3K9 methylation showed a nearly 10-fold loss of compartment interaction strength between H3K27me3 domains (Fig. 2D,E). We conclude that H3K9 methylation is necessary for compartmentalization of H3K27me3 domains.

We wondered whether the requirement for H3K9 methylation was specific for H3K27me3 domains that are normally H3K9me marked in wild type, or alternatively was needed for the compartment structure of both marked and unmarked domains. To investigate this, we separated domains into H3K9me marked and unmarked groups and then assayed compartment and TAD strength in wild-type and *met-2 set-25* mutants. We found that loss of H3K9 methylation strongly reduced the compartment strength of H3K27me3 domains irrespective of whether the domains were marked by any methylated form of H3K9 in wild type (Supplemental Fig. S7C). These results indicate that H3K9 methylation, predominantly found on the distal arms, facilitates the compartment structure of H3K27me3 domains across the chromosomes, even in regions that lack H3K9 methylation. Given the known role of H3K9 methylation in tethering distal chromatin to the nuclear envelope (Towbin et al. 2012), we hypothesize that tethering of distal arm H3K27me3 domains may be important for the compartmental structure of H3K27me3 domains across the autosomal chromosomes.

A parallel studying using Hi-C reported differences in interaction frequencies within the large distal and center autosomal regions in *met-2 set-25* mutants compared to wild type, such as disruption of interactions within the central regions distal arms, which we confirmed using ARC-C (Supplemental Fig. S8; Bian et al. 2020).

Despite the strong effect on H3K27me3 compartment strength, the loss of H3K9 methylation did not have a widespread or specific effect on gene expression in H3K27me3 domains, because *met-2 set-25* mutants displayed similar levels of gene expression dysregulation in active and H3K27me3 domains (Supplemental Fig. S9; Supplemental Table S1). Altered genes were predominantly up-regulated, and gene expression increases were associated with genes normally marked by H3K9 methylation irrespective of whether the gene was located in an active or H3K27me3 domain (Supplemental Fig. S9). We conclude that H3K9 methylation is an important mediator of inactive compartment structure, but that compartment structure itself has little effect on gene expression.

**Figure 3.**
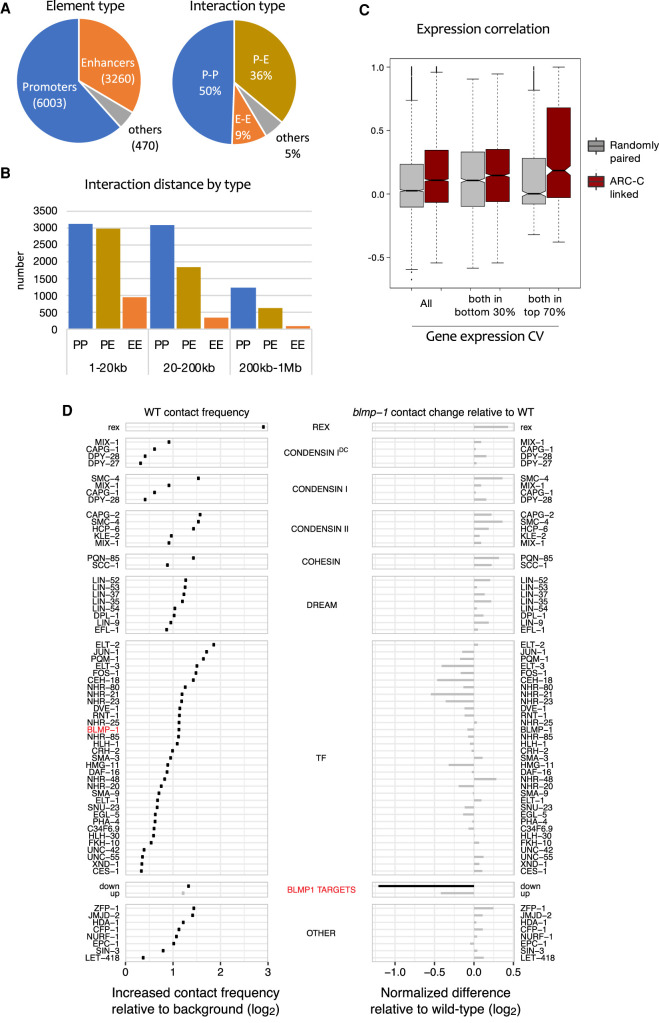
ARC-C defines significant interactions between regulatory elements, candidate regulators, and a role for BLMP-1 in mediating interactions. (*A*) Types of elements and types of interactions involved in the 14,901 significant interactions. (*B*) Interaction distances of the significant interactions, separated by element type. (*C*) Expression correlation between pairs of genes with linked promoters compared to randomly paired genes in the set. (*Left*) All pairs of linked genes (*n* = 5142); (*middle*) pairs for which both genes are in the bottom 30% of CV values (wide expression; *n* = 2224); (*right*) pairs for which both genes are in the top 70% of CV values (regulated expression; *n* = 879). (*D*, *left*) Contact frequency between binding sites of indicated proteins; (*right*) change in contact frequency in *blmp-1* mutants compared to wild type (log_2_). Interactions among *blmp-1* down targets are significantly reduced. See Methods for details and Supplemental Table S5 for data.

We next investigated the ability of ARC-C to profile interactions between regulatory elements. We previously defined 15,714 promoter and 19,231 enhancer elements in *C. elegans* (Jänes et al. 2018). To identify chromatin interactions between these and other genomic elements at high resolution, we separated the genome into 500-bp bins and identified those that interacted significantly with other bins, taking into account distance and coverage biases (Methods). This identified 14,992 chromatin interactions within a distance of 1 kb to 1 Mb (Supplemental Table S2). The interactions involve 9733 different regions, 95% of which overlap an annotated promoter or enhancer (Fig. 3A; Supplemental Table S3). Therefore, ARC-C has the power to map interactions between regulatory elements at 500-bp resolution.

Promoters are most prevalent among the significant interactions, accounting for 62% of interacting elements, and they are involved in 86% of the interactions (Fig. 3A). Half of the significant interactions are relatively short range (within 20 kb), and in this size range we observed a similar number of P–P and P–E interactions (Fig. 3B). However, at longer distances, promoter–promoter interactions predominate. We found that genes connected by promoter–promoter interactions had correlated gene expression (Fig. 3C) and that the correlation is strongest for pairs with highly regulated expression, that is, those with high coefficients of variation of gene expression (CV), suggesting that such genes are in proximity with each other when expressed.

To identify proteins that are candidates for mediating interactions in *C. elegans*, we screened for transcription factors and chromatin regulators for which binding sites show significantly enriched interactions in the ARC-C contact map (Methods). Of 105 proteins tested, 60 chromatin regulators or TFs had this property (Fig. 3D; Supplemental Figs. S10, S11; Supplemental Table S4). Similar assessment of two Hi-C maps (Crane et al. 2015; Brejc et al. 2017) identified only five proteins whose binding sites showed significantly enriched interactions; however, trends were similar to those observed using ARC-C (Supplemental Fig. S10). As expected from the role of the condensin I DC complex in mediating X Chromosome interactions between rex sites (Crane et al. 2015), the binding sites of condensin components were highly enriched for interactions (Fig. 3D). Other proteins of note are cohesin SCC-1 and loading factor PQM-85/NIPBL, consistent with similar enrichment in mammals and with the role of cohesin in loop formation (Rao et al. 2014, 2017); *C. elegans* does not contain an ortholog of boundary factor CTCF, which functions with cohesin in mammals (Heger et al. 2009). We also observed significant enrichment for interactions between binding sites of subunits of the condensin II and Retinoblastoma/DREAM complexes, 33 transcription factors, and other chromatin regulators (Fig. 3D). These proteins are strong candidates for involvement in mediating chromatin interactions.

To evaluate the ability of ARC-C to detect changes in regulatory interactions, we chose to analyze BLMP-1, a TF for which binding sites significantly interact (Fig. 3D). *blmp-1* encodes a transcription factor important for hypodermal, vulval, and gonadal development (Horn et al. 2014; Huang et al. 2014; Yang et al. 2015). We performed ARC-C in L3 stage *blmp-1* mutants and asked whether chromatin interactions at pairs of TF and chromatin regulator binding sites were altered compared to wild-type L3 larvae. No significant changes in interaction frequency were detected for the analyzed factors, including the full set of BLMP-1 binding sites (Fig. 3D; Supplemental Table S5). We next analyzed direct BLMP-1 targets, defined as the subsets of BLMP-1 binding regions associated with a gene that was up-regulated or down-regulated in *blmp-1* mutants (Supplemental Table S6). Chromatin interactions between targets down-regulated in *blmp-1* mutants were significantly reduced (Fig. 3D; down targets in Supplemental Table S5), showing that ARC-C can detect specific changes in mutants. The results suggest that BLMP-1 may promote spatial proximity between targets that require it for expression, for example, by directly mediating interactions. Valid coverage at the down-regulated targets is also reduced 1.2-fold in *blmp-1* mutants, suggesting that BLMP-1 contributes to facilitating open chromatin at these sites.

## Discussion

In its present form, ARC-C works well for profiling chromatin interactions in relatively small genomes, because sequencing 200 million fragments per duplicate library produces enough *cis*-informative read pairs for profiling architecture and regulatory element interactions. For application to larger mammalian genomes, an enrichment step for ligation events (e.g., through biotin tagging) would be beneficial.

Here, we used ARC-C in whole animals, so the cell types from which the detected interactions came are unknown. In addition, interactions that occur in a small number of cells are likely to have been missed. The future application of ARC-C to specific purified cells would address these issues, allowing in vivo investigation of cell type–specific architecture.

In conclusion, ARC-C provides a new ability to study genome topology and regulatory interactions at high resolution in a single genome-wide assay. Our application of ARC-C in *C. elegans* revealed unappreciated domain and compartment structure and proteins that are candidates for mediating organization. The use of ARC-C and study of these candidates should accelerate studies of transcriptional regulation and the relationship with genome architecture.

## Methods

### Worm strains and culture

*C. elegans* strains were maintained at 20°C as previously described (Brenner 1974). The following strains were used: Bristol N2 (wild type), YJ55 *blmp-1(tm548)* (Huang et al. 2014), and GW638 *met-2(n4256) set-25(n5021)* (Towbin et al. 2012).

### Worm growth

Strains for ARC-C and ChIP-seq were grown in liquid culture at 20°C using standard S-basal medium with HB101 bacteria. Animals were first grown to the adult stage, bleached to obtain embryos, and the embryos hatched without food in M9 buffer for 24 h at 20°C to obtain synchronized starved L1 larvae. L1 larvae were grown in a further liquid culture at 20°C then harvested at the L3 stage. Worms were collected, washed in M9 buffer, floated on sucrose, washed again in M9, then frozen into small pellets by dripping worm slurry into liquid nitrogen, which was stored at −80°C until use.

### ChIP-seq

JMJD-2, SCC-1, ZFP-1, BLMP-1, TOP-2, and SIN-3 chromatin immunoprecipitations in L3 larvae and library preparations were conducted as in McMurchy et al. (2017). Antibodies used were JMJD-2 (antibody Q3951; this study, raised against amino acids 50-149), SCC-1 (Novus antibody 29510002, lot Q0835; raised against amino acids 421-520); ZFP-1 (antibody Q2059; this study, raised against amino acids 1-100), BLMP-1 (antibody Q2919; this study, raised against amino acids 43-142), TOP-2 (antibody Q5515; this study, raised against aa317-437), and SIN-3 (antibody Q6013; Beurton et al. 2019).

### Differential expression analysis of *met*-*2 set*-*25* mutant

Wild-type and *met-2 set-25* mutant worms were grown at 20°C on NGM plates, harvested at the L3 stage, and flash frozen in liquid nitrogen. RNA was extracted from frozen worms using TriPure (Roche) and purified with the Zymo Research RNA Clean and Concentrator kit (R1013) after DNase I digestion. Libraries were prepared with the TruSeq RNA Library Prep Kit (Illumina). Reads were aligned to genome assembly ce10 with gene annotation WS235 using STAR (Dobin et al. 2013). Read counts per gene were generated by HTSeq and genes differentially expressed in *met-2 set-25* relative to wild type identified using DESeq2 (Love et al. 2014), requiring adjusted *P* < 0.05 (Supplemental Table S1). Genes were annotated as being marked by H3K9me2 or H3K9me3 if their average signal was 1.5× above the median, using L3 ChIP-seq data sets available under the NCBI Gene Expression Omnibus (GEO; https://www.ncbi.nlm.nih.gov/geo/) accession numbers GSE49728 for H3K9me2 and GSE49720 for H3K9me3 (Ho et al. 2014).

### Differential expression analysis of *blmp*-*1* mutant

Raw RNA-seq data of wild-type and *blmp-1(tm548)* mutants at L3 stage were obtained from GEO (GSE55225) (Horn et al. 2014). Reads were aligned to genome assembly ce10 with gene annotation WS235 using STAR (Dobin et al. 2013). Read counts per gene were generated by featureCounts, and genes differentially expressed in *blmp-1* relative to wild type were identified using DESeq2 (Love et al. 2014), requiring adjusted *P* < 0.05 and absolute fold change >1.5. Genes significantly up-regulated or down-regulated in *blmp-1* mutants compared to wild type and for which a BLMP-1 ChIP-seq peak overlapped one of its assigned regulatory elements were defined as up-regulated “up” or down-regulated “down” BLMP-1 targets (Supplemental Table S6).

### ARC-C library preparation

A key element of ARC-C is to digest nuclei with DNase I in situ under conditions in which cutting results in maximal enrichment of informative read pairs at accessible chromatin. This concentration should be empirically determined for the sample type of interest. For *C. elegans* nuclei, we found that 50–200 units/mL DNase I gives optimal recovery of informative read pairs using the digestion conditions indicated below (Supplemental Fig. S1).

Frozen worm pellets were ground into a fine powder in which worms were broken into approximately 10 fragments. Then 1 mL of worm powder was fixed in 10 mL of 1% formaldehyde in PBS for 10 min at room temperature (RT) with gentle shaking then quenched for 5 min with a final concentration of 125 mM glycine. Fixed worm fragments were then washed with Buffer A (340 mM sucrose, 15 mM Tris-HCl at pH 7.5, 2 mM MgCl_2_, 0.5 mM spermidine, 0.15 mM spermine, 1 mM DTT, protease inhibitors), resuspended in 7 mL Buffer A, then the material dounced 20 strokes in a 7 mL stainless steel tissue grinder (VWR 432-5005). The dounced material was spun at 100*g* for 5 min; the supernatant, which contains nuclei, was transferred to a new tube. The pellet was resuspended in Buffer A and again dounced with 20 strokes. After spinning, the two supernatants containing nuclei were pooled.

Aliquots of 10 million fixed nuclei were spun down at 1000*g* and resuspended in 200 µL of 1× DNase buffer (Roche), and chromatin was digested with 50 and/or 100 units/mL DNase I for 10 min at 25°C. The reactions were then quenched with a final concentration of 25 mM EDTA and 5 mM Tris at pH 7.5. Nuclei were washed twice with 1 mL of ice-cold Nuclear Washing Buffer (340 mM sucrose, 15 mM Tris-HCl at pH 7.5, 25 mM EDTA, 0.5 mM spermidine, 0.15 mM spermine, 1 mM DTT, protease inhibitors). Nuclei were then resuspended in 100 µL of end repair master-mix (10 µL 10× NEB End Repair Buffer, 5 µL NEB End Repair Enzyme Mix, 85 µL H_2_O) and incubated for 30 min at 20°C with rotation. Thereafter, 400 µL of ligation master-mix was added (40 µL 10× ligation buffer, 5 µL T4 DNA ligase [400,000 units/mL], 355 µL H_2_O) and the mixture was incubated overnight at 4°C with rotation. Nuclei were pelleted, resuspended in 50 µL of tagmentation master-mix (22.5 µL H_2_O, 25 µL 2× Nextera TD buffer, 2.5 µL Nextera Tn5 transposase), and incubated for 30 min at 37°C. Next, 5 µL of 1% SDS and 2 µL of NEB Proteinase K [800 units/mL] was added and left for 15 min at 65°C before DNA from the mixture was purified using Qiagen MinElute columns. Large fragments (>500 bp) were removed from the purified DNA using two rounds of 0.6vol AMPure XP beads. Before PCR amplification, a test qPCR amplification using 1/20th of the input DNA was performed, and the cycle number for PCR was determined by the midpoint of the exponential phase of the amplification curve. The resultant DNA was amplified with NEBNext Ultra II Q5 Master-mix under the following PCR conditions: for 5 min at 72°C, for 30 sec at 98°C, and cycling for 10 sec at 98°C, for 30 sec at 63°C, and for 1 min at 72°C using the determined cycle number. The library was size-selected with AMPure XP beads to a final range of 200–700 bp (insert size: ∼70–570 bp). Before sequencing, libraries were quality tested by qPCR at the *gap-3* promoter. DNase I overdigestion or underdigestion led to poor enrichment of regulatory elements. Good libraries generally had *gap-3* promoter enrichment values of greater than fourfold. Libraries were sequenced on the Illumina platform paired-end (100 bp). Four libraries were sequenced from wild-type L3 larvae (three biological replicates and two technical replicates; separate ARC-C libraries made from the same biological material: N2-rep1, N2-rep2a, N2-rep2b, and N2-rep3 in Supplemental Fig. S2A), and two biological replicate libraries were sequenced for both *blmp-1* and *met-2 set-25* L3 larvae.

### Processing ARC-C data

ARC-C libraries were sequenced 62–150 bp (100 bp for most samples) from both ends. Supplemental Figure S2 lists libraries analyzed in the paper and sequencing statistics. Adapter sequence was trimmed by cutadapt (Martin 2011), and sequences with >20 bp remaining were removed. Each sequenced end was aligned independently to the ce10 reference genome using BWA-MEM (Li 2013), which allows split-read alignment using the default parameters. The two aligned ends (or the 5′ segment for split alignments) were then paired. We required both ends of a pair to align uniquely and with high confidence (mapping quality ≥ 30 and number of mismatches ≤ 2) to the nuclear genome and outside modENCODE backlisted-regions. PCR duplicates were next removed by sambamba markdup. The remaining read pairs were regarded as *valid* read pairs. Valid read pairs mapping to different chromosomes or >600 bp apart on the same chromosome were regarded as *trans-* or *cis-informative* read pairs, respectively. The 600-bp threshold was established by comparing the proportions of the four possible end alignment orientation configurations (forward–forward, forward–reverse, reverse–forward, and reverse–reverse) as a function of mapping distance. The vast majority of pairs mapping <500 bp apart were in the forward–reverse configuration (nonligated fragments), whereas >600 bp the proportions were stably at ∼25% each (Supplemental Fig. S12).

Contact maps were made from informative read pairs by binning the genome into fixed-width (1, 5, 10, or 50 kb) nonoverlapping bins and counting the number of read pairs between each pair of bins. The maps were then normalized by matrix balancing using the Knight–Ruiz algorithm (Knight and Ruiz 2013). Concordance between replicate maps was assessed by GenomeDISCO (Ursu et al. 2018). The lower resolution 50-kb maps were used for whole-chromosome visualization. The 1-, 5-, and 10-kb maps were further corrected for distance-dependent background contact frequency by dividing the spline-smoothed average contact frequency given the distance to the diagonal. These higher resolution maps were used for aggregated contact analysis (see below) of nuclear factor binding sites and chromatin domain/compartment, respectively.

### Processing Hi-C data

Raw FASTQ files of wild-type mixed embryo Hi-C data (Crane et al. 2015) were downloaded from the NCBI Sequence Read Archive (SRA; https://www.ncbi.nlm.nih.gov/sra) accession number SRX77040 and processed using HiCUP v0.5.9 (Wingett et al. 2015), which filters for same fragment (circularized, dangling ends, internal), religation, wrong size, contiguous sequence, and removes duplicate read pairs. We additionally required mapQ ≥ 30 and number of mismatches ≤2 from both reads, that none of the reads overlapped modENCODE blacklisted regions, and a minimum distance of 600 bp between the two read pairs to be consistent with the processing of ARC-C data. In the end, 25,460,294 read pairs passed all filters, of which 17,100,808 have both reads mapping to the same chromosome.

### Calling significant interactions

We first segmented the genome into bins of ∼500 bp using the following procedure. We first took annotated regulatory elements (Jänes et al. 2018) (*n* = 42,245) and expanded them to 500 bp or until neighboring intervals began to touch; a small number of elements that were within 100 bp were merged first. The rest of the genome was covered with evenly placed 500-bp nonoverlapping fixed-width intervals; hence, the entire genome was covered by a combined set of 192,257 intervals of average size (494 bp). We used this procedure instead of generating fixed nonoverlapping bins to avoid individual Res being split into two bins.

Before assessing the significance of chromatin interactions in Hi-C or other chromatin interaction data sets, inherent bias resulting from uneven coverage and physical proximity need to be accounted for. A difference in ARC-C data compared to that of Hi-C is the specific overrepresentation of open chromatin. The observed enrichments at these regions are a compound effect of enriching the regions themselves and of their being enriched by virtue of linkage to another region of open chromatin (true signals). To address this and avoid normalizing out interaction signals, we developed an approach similar to that used for Capture Hi-C, which has similar coverage biases and used “off-peak” interaction frequency to account for coverage (Cairns et al. 2016). We modeled the number of read pairs linking two intervals as a random variable following a binomial distribution parameterized by an expected contact frequency determined by unevenness of coverage and distance between the interval (Ay et al. 2014), as described next.

For every interval *i*∈*I*, the number of *cis*-informative read pairs *c*_*cis*,*i*_ were counted. Intervals in the top 10% of the coverage distribution were regarded as peaks, and intervals in the bottom 10% were removed. An off-peak *cis*-informative coverage *c*_offpeak,*i*_ was calculated for every kept interval, counting the number of contacts not involving peak intervals. We calculated a scaling factor for the interval's representation/visibility as *v*_*i*_ = (*c*_offpeak,*i*_/median(*c*_offpeak,._))^0.87^ (see below for derivation of the exponent). Chromosome-wide average distance-dependent contact frequency *F*(*d*) in the distance range of 1 kb to 1 Mb was modeled by fitting a spline function in a two-pass process (following Fit-Hi-C) (Ay et al. 2014). For every pair of intervals with a distance between 1 kb and 1 Mb, an expected contact frequency was calculated given the distance and the visibility of each interval as *f*_*i*,*j*_ = *v*_*i*_ *v*_*j*_ *F*(*d*_*i*,*j*_). Given the total number of *cis*-informative contacts (*N*) of the chromosome, we considered a null distribution in the form of a binomial, where the observed number of contacts, *n*_*i*,*j*_ ∼ binomial(*N*, *f*_*i*,*j*_). Significant interactions were called at an FDR level of 0.05 and were post-filtered requiring support by more than five read pairs.

The appropriate correction factors for adjusting the representation bias, *X*, should be able to transform the unnormalized contact matrix *A* into a normalized contact matrix *B* by *B* = *diag*(*X*)*A*diag(*X*), such that each row or column of *B* sums up to the same value, thus eliminating the unevenness of representation across different bins. It has been well-established that a correct set of factors can be found using the method “matrix balancing” (MB) (Imakaev et al. 2012; Rao et al. 2014), and an efficient algorithm has been developed (Knight and Ruiz 2013; Rao et al. 2014). Our goal is to find correction factors that enable transformation into a normalized contact matrix in which each bin has the same off-peak coverage. Because an efficient algorithm for solving this problem at high resolution is not yet available, we aimed to find an approximate solution. Two measures, namely, the reciprocal of coverage (*c*) and square root coverage (*c*^0.5^), have been proposed for use in the place of MB-derived correction factors (Rao et al. 2014). It was reported that the former can overcorrect, whereas the latter gives good approximation to MB-derived correction factors (Rao et al. 2014). We examined correlation between MB-derived correction factors and the reciprocal of different exponents of coverage (Supplemental Fig. S13) and found that the reciprocal of *c* indeed overcorrects, but the reciprocal of square root coverage undercorrects. The most accurate approximation is achieved at around an exponent of 0.87. Therefore, we used the reciprocal of (*c*_offpeak_)^0.87^ as the correction factor.

### Aggregated contact analyses

A contact is defined as the region in the contact map that connects a pair of genomic locations. Aggregated contact analysis is a method of visualizing the average contact frequency of a group of many contacts together with local contact frequency (Rao et al. 2014). We applied this method to both small genomic regions, such as nuclear factor ChIP-seq binding sites (NFBS), and to larger intervals, such as chromatin domains. We normalized contact maps using matrix balancing (Imakaev et al. 2012) to account for coverage bias and removed distance-dependent background. In NFBS analysis, we used normalized and background-frequency-corrected contact maps of 1-kb resolution. For each NF, up to 50,000 contacts were randomly sampled from all possible *cis*-contacts among its binding sites within a distance range from 20 kb to 1 Mb, and local maps of 21 × 21 bins centered at the contacts were extracted and aggregated. For the case of BLMP-1 regulated targets, all possible *cis*-contacts between BLMP-1 binding sites that involves a BLMP-1 regulated target were aggregated (i.e., at least one end of the contact is at a BLMP-1 regulated target). The log_2_ fold change of the central point over the mean of the rest of the points in the aggregated map was calculated to measure the relative increase in contact frequency over local background. To assess statistical significance while controlling for accessibility and the distance between the pair of NFBSs, 1000 sets of random contacts were generated, each containing the same number of contacts with matching accessibility and distance as the NFBS contacts. Each of the 1000 random sets was aggregated and a relative increase in contact frequency was calculated in the same way as the NFBS set, forming a distribution of values against which the NFBS value was compared and a *P*-value generated, which was corrected for multiple testing using the FDR method. The rex–rex APA analyses used normalized and background-frequency-corrected contact maps of 10-kb resolution and a distance range of 100 kb to 4 Mb. Data sets (Kranz et al. 2013; Araya et al. 2014; Ho et al. 2014; Latorre et al. 2015; Wiesenfahrt et al. 2016; McMurchy et al. 2017; Kudron et al. 2018) are listed in Supplemental Table S7. Processed and curated modENCODE ChIP-seq peaks (Araya et al. 2014; Kudron et al. 2018) were obtained from Jänes et al. (2018). For ChIP-seq performed in this paper (JMJD-2, SCC-1, ZFP-1, BLMP-1, TOP-2, and SIN-3), peaks were called for each replicate separately as for accessible sites in Jänes et al. (2018) using YAPC (https://github.com/jurgjn/yapc), except for BLMP-1, which used MACS2 (Feng et al. 2012). ChIP-seq peaks were combined by IDR (Li et al. 2011) with a *P*-value threshold of 0.01. We removed intervals that are highly occupied target (“HOT”) because such binding events are thought to represent non-sequence-specific TF binding or ChIP artifacts (Gerstein et al. 2010; Kudron et al. 2018). This was defined as the top 20% of peak intervals ranked by the number factors in which the interval is called (effectively removing intervals called in 12 or more factors). We only considered data sets having at least 300 peaks following filtering.

For domain analyses, we used normalized and background-frequency-corrected contact maps of 5-kb resolution. L3 stage chromatin domains are from Evans et al. (2016). Regions annotated as active and border were merged to generate the active domains used here; the H3K27me3 domains are those termed “regulated.” Informative reads mapped at similar levels to active and H3K27me3 domains: 10.6 million (44.4%) of all informative reads mapped to active domains (39.0 Mb, 38.9% of the genome), whereas 8.1 million (33.8%) mapped to H3K27me3 domains (42.3 Mb, 42.1% of the genome). The rest of the reads mapped to Chromosome X, for which active and H3K27me3 domains were not mapped. The small (1.42-fold) bias in coverage was normalized by the matrix balancing that was performed on the contact frequency matrix to normalize the differences in sequencing coverage across all genomic bins.

We tested for TADs by assessing intra-domain contacts. For each type of domain (active or H3K27me3), maps of each contact region containing a domain of at least 5 kb together with up to 25 kb of the flanking domains of opposite type were extracted and aggregated. Where neighboring domains were <25 kb, only the domain was extracted. The aggregated contact was scaled to a square of 9 × 9 bins, and the flanking intervals were scaled to five bins wide. The log_2_ fold change of the mean of the central 9 × 9 square over the mean of the four neighboring 5 × 10 rectangles on top, bottom, left, and right was calculated to measure the relative strength in contact frequency with *P*-values generated by *t*-test. We tested for compartments using the same approach, by assessing all possible pairs of inter-domain contacts in the range from 50 kb to 2 Mb. To compare domain and compartment strength in *met-2 set-25* mutants relative to wild type, the fold change in percentage difference between central block and side blocks across strains were calculated.

To test the effect of local H3K9 methylation on TAD and compartment strength, we separated active and H3K27me3 domains into highly and lowly marked sets. For each active and H3K27me3 domain, the average coverage of H3K9me1, H3K9me2, and H3K9me3 was calculated using wild-type L3 ChIP-seq data (Ho et al. 2014). For each modification, active and H3K27me3 domains in the top 25% of signal were defined as H3K9me1, H3K9me2, or H3K9me3 high domains, and the remainder as low domains. Aggregated contact analysis of TADs and compartments was performed using wild-type and *met-2 set-25* ARC-C data for each of the domain sets as described above.

### Aggregated contact analysis of *blmp*-*1* mutant ARC-C data

We used the procedure described above for wild type to measure the relative contact frequency between NFBSs in *blmp-1* ARC-C data. To normalize overall open chromatin measurements between *blmp-1* and wild-type maps, we first fitted *blmp-1* ACA measures as a linear function of the respective measures in wild type, that is, log_2_(blmp1_FC) = a + b * log_2_(N2_FC). The residuals were then used to measure the difference in contact frequency in *blmp-1* relative to wild type. Statistical significance was assessed by 10,000-time bootstrapping the distribution of residuals, and *P*-values were adjusted by FDR (Supplemental Table S5).

### Expression correlation between interacting promoters

For genes linked by promoter–promoter interactions, we calculated Pearson correlation coefficients of gene expression across cell types using data from Cao et al. (2017). For pairs involving bidirectional promoters, the gene pair with highest correlation was chosen. For each gene, we also calculated a coefficient of variation of gene expression (CV) across the cell types (Cao et al. 2017) as a measure of tissue-biased expression. Genes with similar expression across cell types have low gene expression CV values and those with tissue-biased expression have high CV values. In Figure 3C, we assessed expression correlation between all linked pairs of genes (*n* = 5124), linked genes for which both were in the bottom 30% of all CV values (*n* = 2224), and linked genes for which both were in the top 70% of CV values (*n* = 879). To assess the statistical significance of the correlations, we generated control sets by sampling the same number of random pairs using genes from the respective linked gene sets while requiring the distribution of distance between the random pairs to match that of the observed set. Difference in correlation between the observed and the random control set was tested using a *t*-test: all (*P* = 7.77 × 10^−27^), bottom 30% (*P* = 4.38 × 10^−06^), and top 70% (*P* = 5.69 × 10^−20^).

## Data access

The ARC-C, ChIP-seq, and RNA-seq data generated in this study have been submitted to the NCBI Gene Expression Omnibus (GEO; https://www.ncbi.nlm.nih.gov/geo/) under accession number GSE144673. The code used in this paper is available at GitHub (https://github.com/nh3/ctk) and as Supplemental Code.

## Supplementary Material

Supplemental Material
