# Supplementary material for: Accessible Region Conformation Capture (ARC-C) gives high-resolution insights into genome architecture and regulation
Source: Genome Res. 2022 Feb;32(2):357–66. doi: 10.1101/gr.275669.121 (PMC8805715; doi:10.1101/gr.275669.121)
Supplement: Supplemental Material [file supp_32_2_357__DC1.html]

Accessible Region Conformation Capture (ARC-C) gives high-resolution insights into genome architecture and regulation — Supplemental Material 

# Accessible Region Conformation Capture (ARC-C) gives high-resolution insights into genome architecture and regulation

## Supplemental Material

- Supplemental\_TableS1.xlsx
- Supplemental\_TableS2.xlsx
- Supplemental\_TableS3.xlsx
- Supplemental\_TableS4.xlsx
- Supplemental\_TableS5.xlsx
- Supplemental\_TableS6.xlsx
- Supplemental\_TableS7.xlsx
- Supplementalcode.zip
- SupplementalFigures\_JDedit.pdf
