# Supplementary material for: Accessible Region Conformation Capture (ARC-C) gives high-resolution insights into genome architecture and regulation
Source: Genome Res. 2022 Feb;32(2):357–66. doi: 10.1101/gr.275669.121 (PMC8805715; doi:10.1101/gr.275669.121)
Supplement: Supplemental Material [file supp_gr.275669.121_SupplementalFigures_JDedit.pdf]

A DNase I concentration (U/ml)

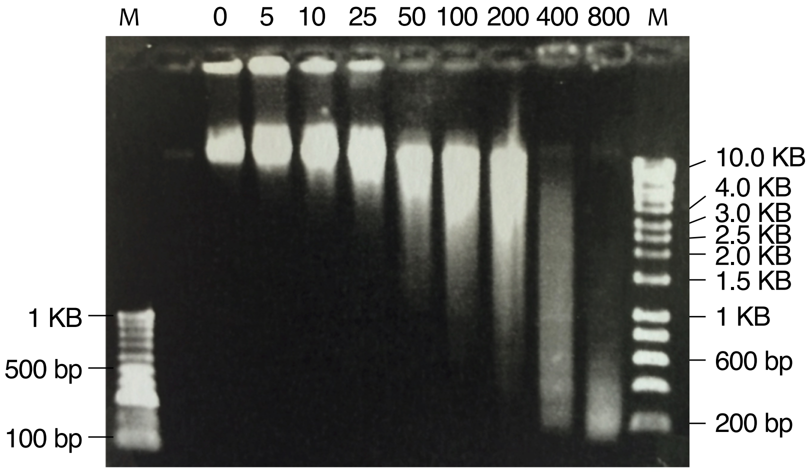

B

DNase I concentration (U/ml)	Enrichment of cis-informative coverage over accessible sites	Percent informative reads
5	1.3	1.22%
10	1.5	1.98%
25	2.2	4.27%
50	3	7.12%
100	3.7	11.85%
200	2.8	11.32%

Supplemental Fig. S1 Empirical determination of DNAse I concentration for optimal recovery of interactions between accessible chromatin sites.

(*A*) Genomic DNA digestion patterns resulting from different concentrations of DNase I. (*B*) Enrichment of cis-informative reads at accessible sites relative to background and percent informative reads for ARC-C libraries prepared after digesting with different concentrations of DNAse I. Accessible sites are from (Janes et al. 2018).

A

Sequencing statistics

	N2-rep1	N2-rep2a	N2-rep2b	N2-rep3	*blmp-1* -rep1	*blmp-1* -rep2	*met-2 set-25* -rep1	*met-2 set-25* -rep2
Total reads	395166552	261529124	263781134	227273986	171056366	92794660	199043186	318798182
mapped	339865256	226265095	241537115	218276630	90439059	36837378	164782322	236147255
paired mapq30	245647846	159352418	180531150	151284678	54101084	25695040	101590260	125117274
valid reads	109874032	88975324	134004104	80576176	35616242	23229198	69153378	78544870
informative reads	9271402	7350866	8047186	6068794	3973496	2434804	5653462	6206544
cis-informative	7212418	5731300	6262410	4760748	2668008	1600316	4476458	4594104
cis-ratio	0.7779	0.7797	0.7782	0.7845	0.6715	0.6573	0.7918	0.7402

B

Genome Disco reproducibility at 20kb resolution

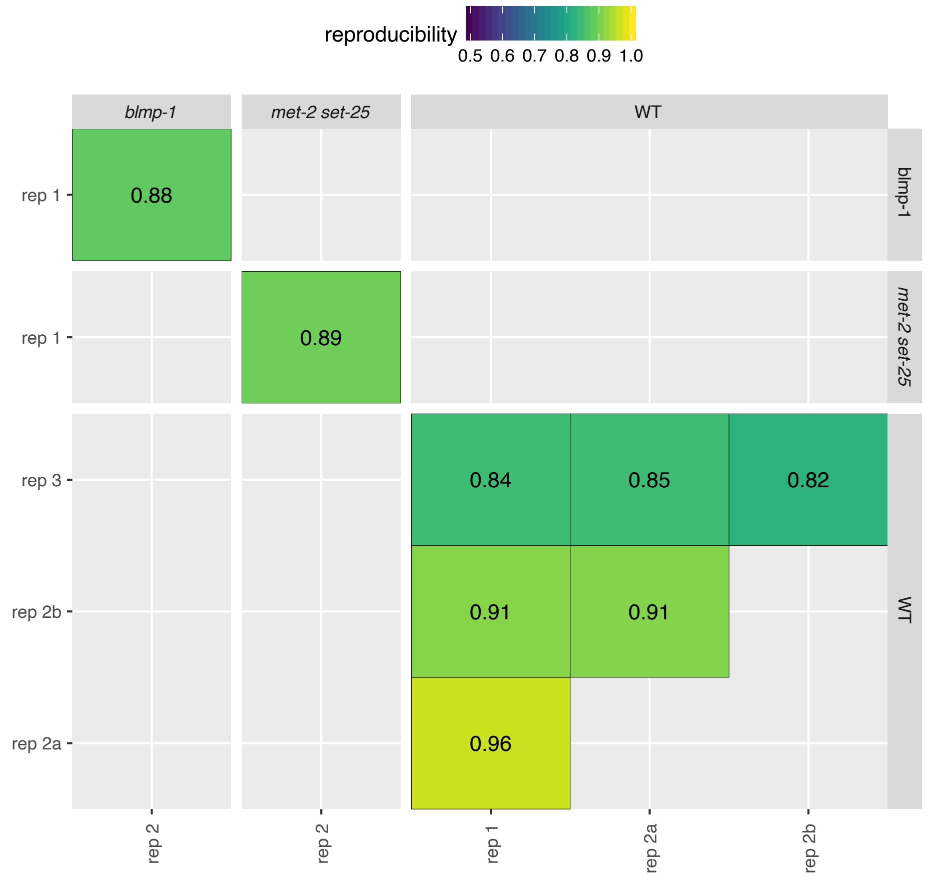

Supplemental Fig. S2 Sequencing statistics and replicate correlations. (*A*) Sequencing statistics of reads for each replicate. **(***B***)** GenomeDicso (Ursu et al. 2018) reproducibility between replicates using 20 kb binned data.

Library	Reference	Unique reads	Deduplicated reads	Informative reads	% Informative reads (relative to uniquely mapped reads)	% Informative reads (relative to deduplicated reads)
ARC-C *C. elegans* WT	this paper	736,816,092	413,429,636	30,738,248	4.17	7.43
ARC-C *blmp-1*	this paper	79,796,124	58,845,440	6,408,300	8.03	10.89
ARC-C *met-2 set-25*	this paper	226,707,534	147,698,248	11,860,006	5.23	8.03
**Studies that assayed regulatory interactions**						
Targeted DNase Hi-C H1 (pe)	Ma et al 2015	66,661,520	56,158,312	1,081,064	1.62	1.93
Targeted DNase Hi-C K562 (pe)	Ma et al 2015	79,970,100	47,904,586	1,411,592	1.77	2.95
Capture Hi-C CD34	Mifsud et al 2015	138,217,259	48,087,767	12,685,127	9.18	26.38
Capture Hi-C GM12878	Mifsud et al 2015	135,546,709	54,397,117	10,532,346	7.77	19.36
Capture-C mESC	Hughes et al 2014	127,817,852	69,226,329	259,350	0.20	0.37
Capture-C Ter119+ rep 1	Hughes et al 2014	70,108,284	33,388,250	376,730	0.54	1.13
Capture-C Ter119+ rep 2	Hughes et al 2014	262,887,176	97,528,819	419,695	0.16	0.43
***C. elegans* Hi-C studies**						
Hi-C *C. elegans* WT	Brejc et al 2017	647,306,152	563,816,513	265,931,063	41.08	47.17
Hi-C C. elegans WT	Crane et al 2015	333,826,368	247,125,231	137,443,864	41.17	55.62

Supplemental Fig. S3 Comparison of the efficiency of ARC-C to other chromatin interaction methods

Efficiencies of ARC-C (top), methods that map regulatory interactions (middle) and *C. elegans* Hi-C studies (bottom). Informative reads are those used for downstream analyses in the individual studies, following authors' filtering for potential artefacts.

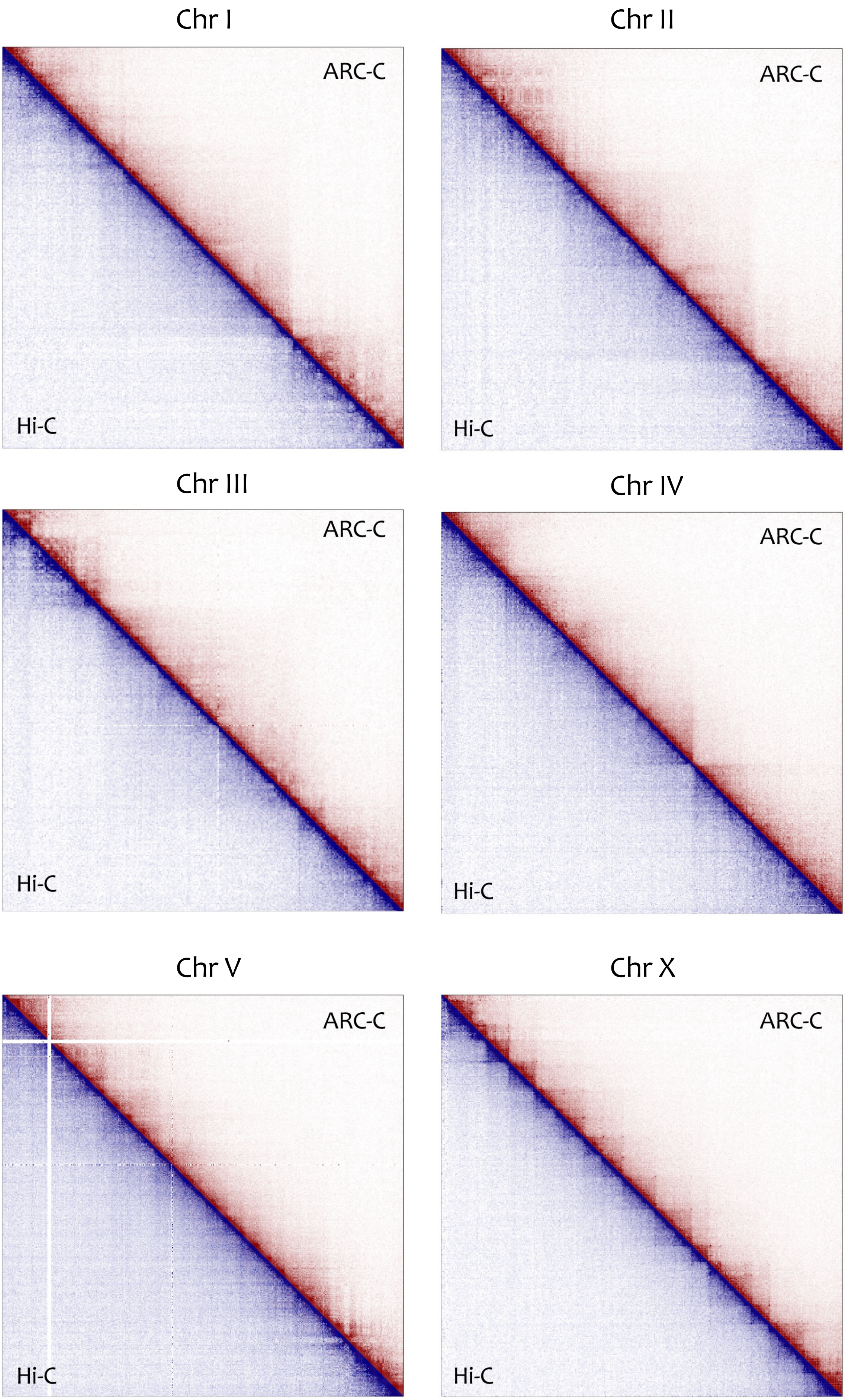

Supplemental Fig. S4 Comparison of ARC-C and Hi-C contact maps for each of the six *C. elegans* chromosomes at 50 kb resolution. Hi-C data are from Crane et al. 2015.

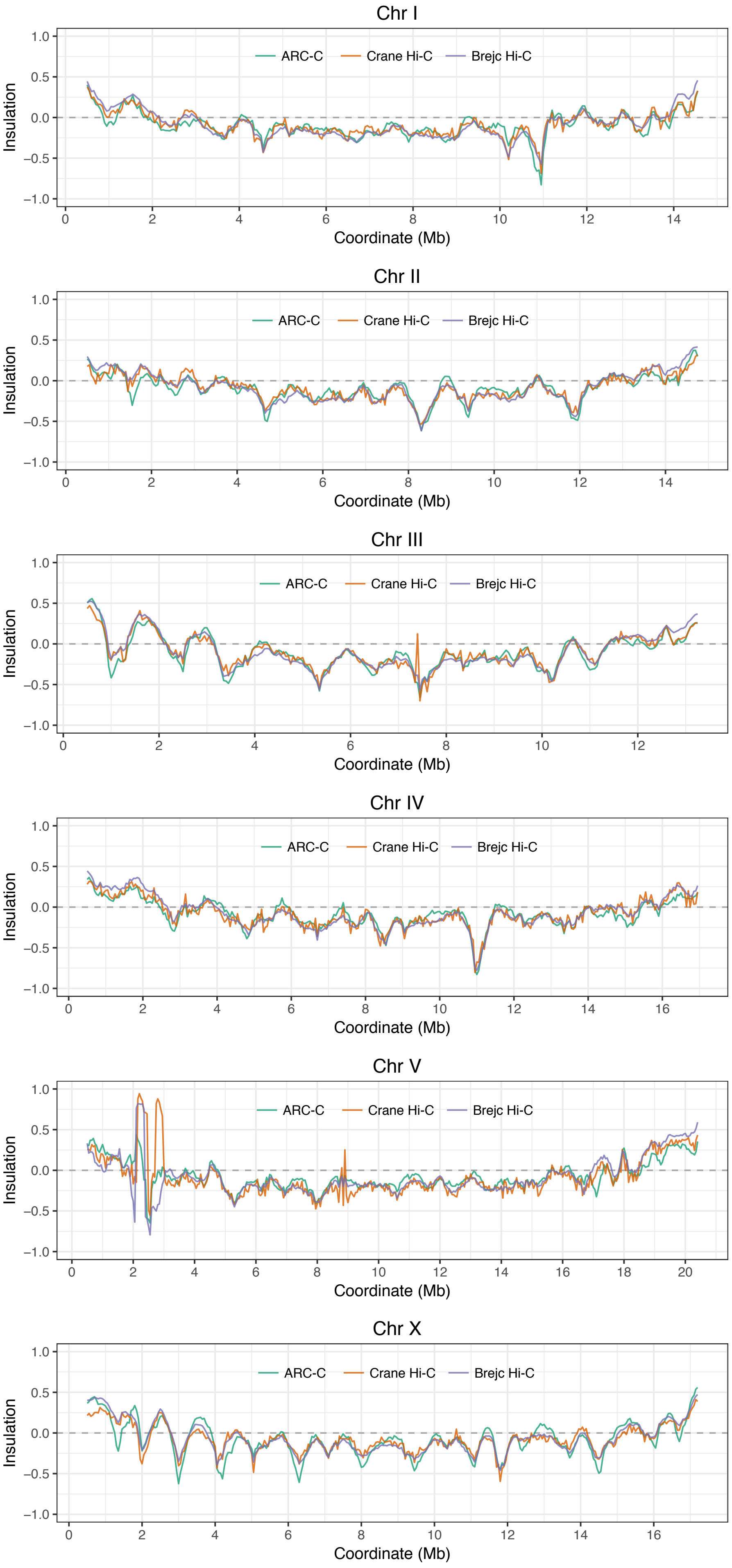

Supplemental Fig. S5 Comparison of whole chromosome insulation plots derived from ARC-C or Hi-C data

Comparison of insulation scores between ARC-C (this study) and two Hi-C maps (Crane et al. 2015; Brejc et al. 2017) calculated with a 500kb sliding block as in Crane et al. 2015.

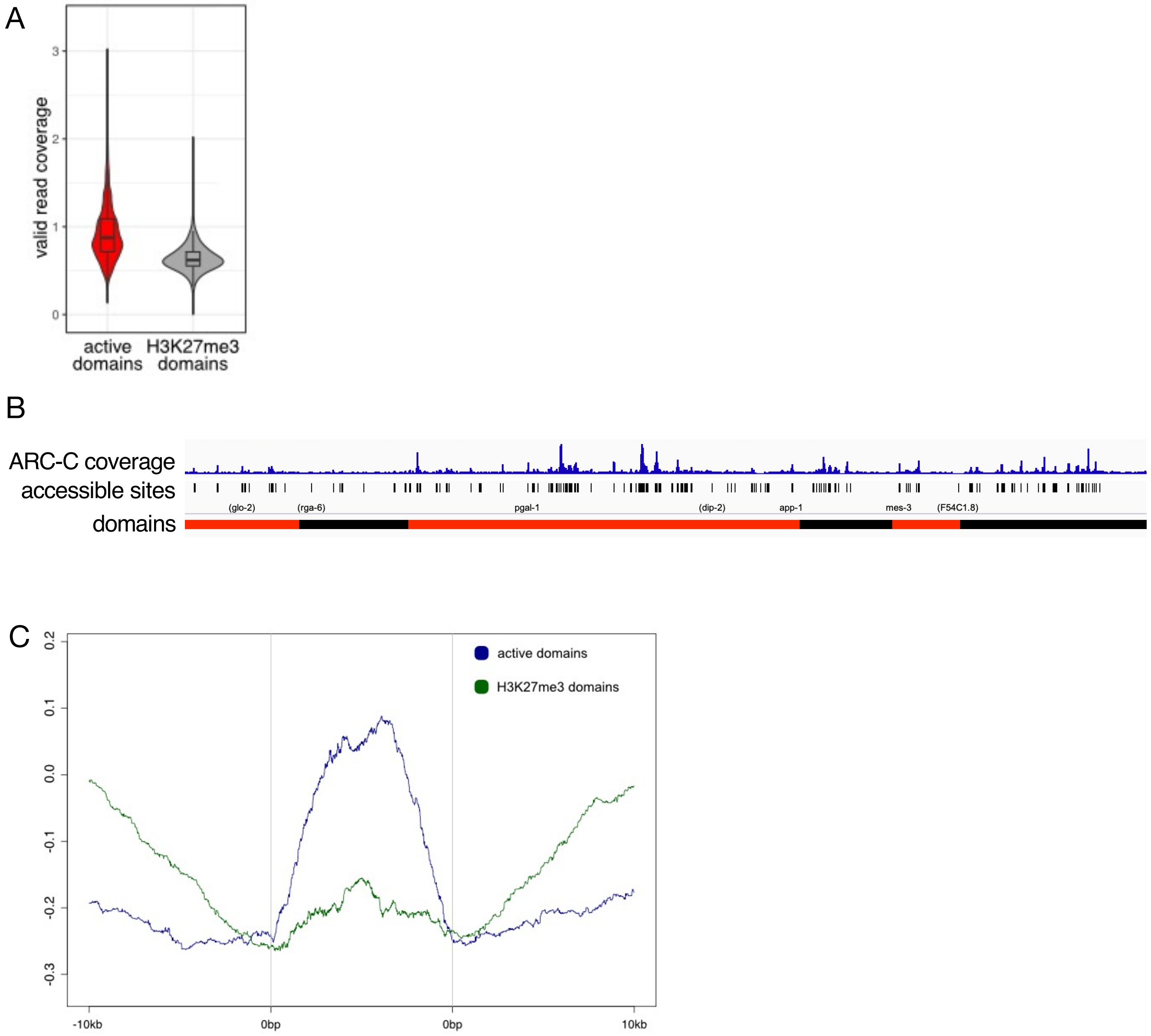

Supplemental Fig. S6 ARC-C coverage across active and H3K27me3 domains

(*A*) Violin plots showing distribution of valid coverage signal across active and H3K27me3 domains. (*B*) IGV screen shot of valid reads across a region of Chr IV. Accessible sites are from (Janes et al. 2018). (*C*) Median insulation scores at 1kb resolution with a 10kb sliding block calculated as in Crane et al. 2015 were plotted across aggregated pseudoscaled active or H3K27me3 domains and the associated 10kb upstream and downstream genomic regions.

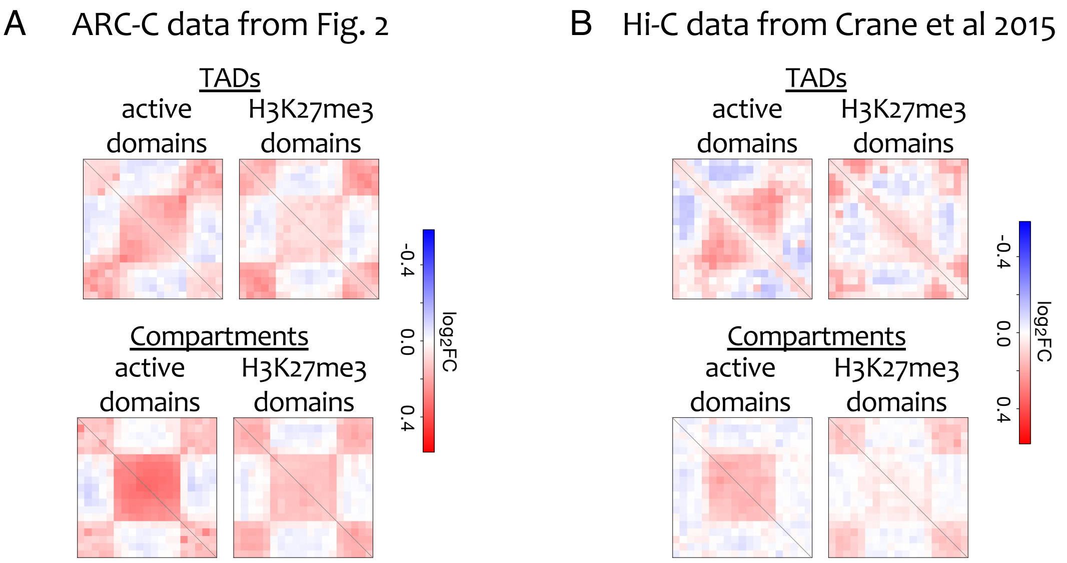

C Compartment strength by H3K9 methylation

All domains

Domain type	Fold enrichment in wt	p-value	Fold enrichment in *met-2 set-25*	p-value
active	1.23	2.786E-195	1.23	2.8825E-90
regulated	1.10	1.253E-45	1.01	0.36080188

Domains separated by H3K9me3 marking

	H3K9me3 +				H3K9me3 -			
Domain type	Fold enrichment in wt	p-value	Fold enrichment in *met-2 set-25*	p-value	Fold enrichment in wt	p-value	Fold enrichment in *met-2 set-25*	p-value
active	1.24	1.6615E-14	1.24	5.0665E-08	1.22	2.639E-164	1.22	7.1357E-75
H3K27me3	1.12	9.5572E-19	1.03	0.16230676	1.10	3.4635E-27	1.01	0.56483504

Domains separated by H3K9me2 marking

	H3K9me2 +				H3K9me2 -			
Domain type	Fold enrichment in wt	p-value	Fold enrichment in *met-2 set-25*	p-value	Fold enrichment in wt	p-value	Fold enrichment in *met-2 set-25*	p-value
active	1.15	4.2646E-23	1.17	5.9601E-13	1.23	1.187E-149	1.23	7.5875E-68
H3K27me3	1.12	6.7409E-11	1.03	0.36247315	1.09	9.4308E-28	1.01	0.58908199

Domains separated by H3K9me1 marking

	H3K9me1 +				H3K9me1 -			
Domain type	Fold enrichment in wt	p-value	Fold enrichment in *met-2 set-25*	p-value	Fold enrichment in wt	p-value	Fold enrichment in *met-2 set-25*	p-value
active	1.20	1.1858E-54	1.22	9.2205E-29	1.21	2.7876E-95	1.20	2.1015E-41
H3K27me3	1.08	0.00606599	1.03	0.6075745	1.10	2.0856E-36	1.02	0.20855563

Supplemental Fig. S7 TAD and compartment analyses using ARC-C and Hi-C data, and ARC-C analyses in active and h3K27me3 domains separated by H3K9me marking

(*A*) ARC-C plots reproduced from Fig 2. Top: TAD enrichment is 1.12 fold for active domains and 1.06 fold for H3K27me3 domains. Bottom: compartment enrichment is 1.23 fold for active domains and 1.10 fold for H3K27me3 domains. (*B)* Plots are as in (a) but using Hi-C data from Crane et al. 2015. Top: TAD enrichment is 1.1 fold for active domains and 1.04 fold for H3K27me3 domains. Bottom: compartment enrichment is 1.11-fold for active domains and 1.03 for H3K27me3 domains. (*C*) Enrichment for compartment interactions in active and H3K27me3 domains in wild-type and met-2 set-25 mutants separated by H3K9 methylation marking; p-values give the significance of the enrichment relative to background. Red values indicate conditions for which there is no significant enrichment for compartment interactions. See Fig 2 and methods for details.

Supplemental Figure S8

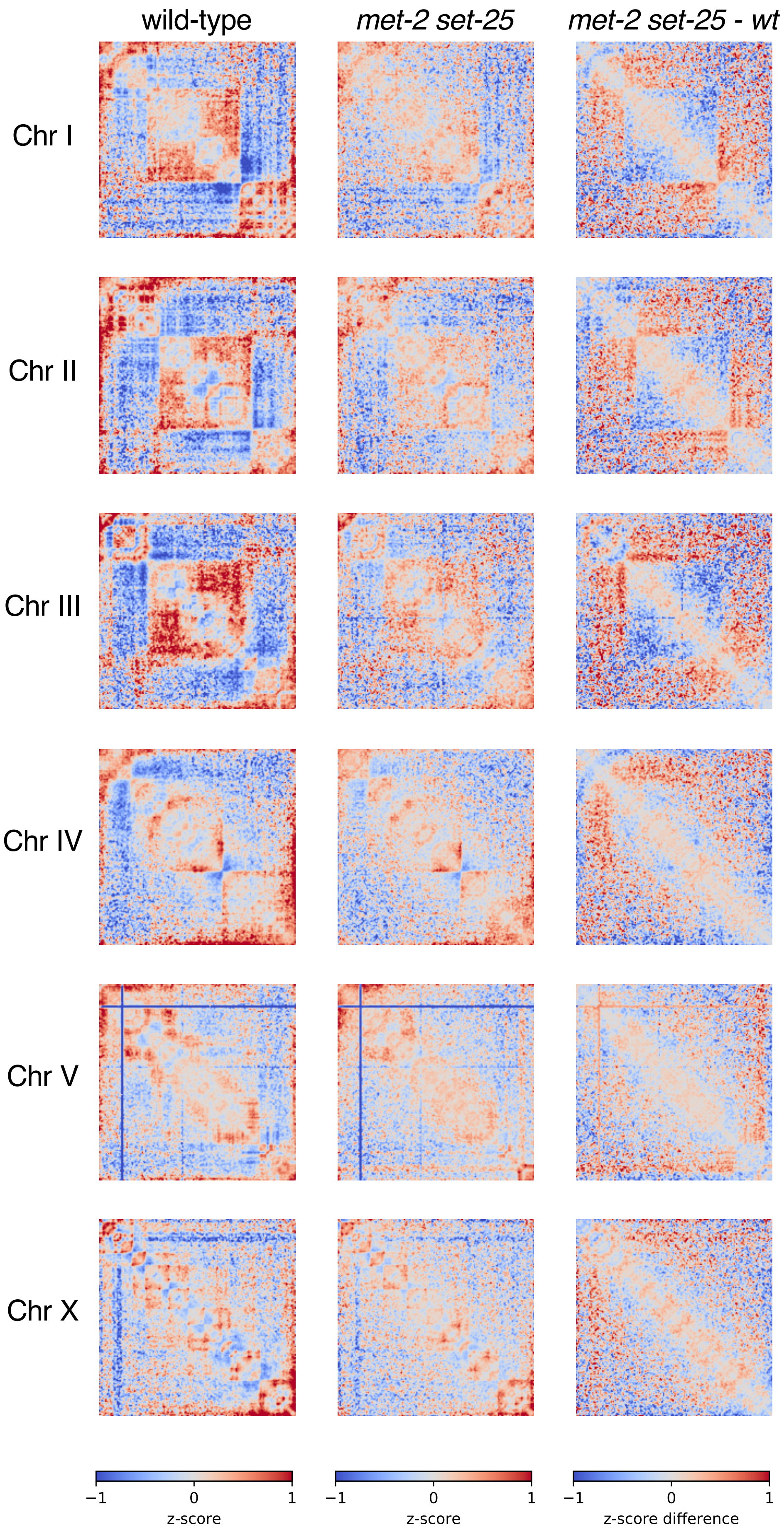

Supplemental Fig. S8 Comparison of ARC-C contact maps in wild-type and *met-2 set-25* mutants

(left, middle) Whole chromosome z-score heatmap shows higher (red) or lower (blue) than expected contact frequency in wild-type (left) and *met-2 set-25* mutants (middle). (right) Whole chromosome z-score subtraction heatmap shows increased (red) or decreased (blue) contact frequency in *met-2 set-25* compared to wild-type.

Supplemental Figure S9

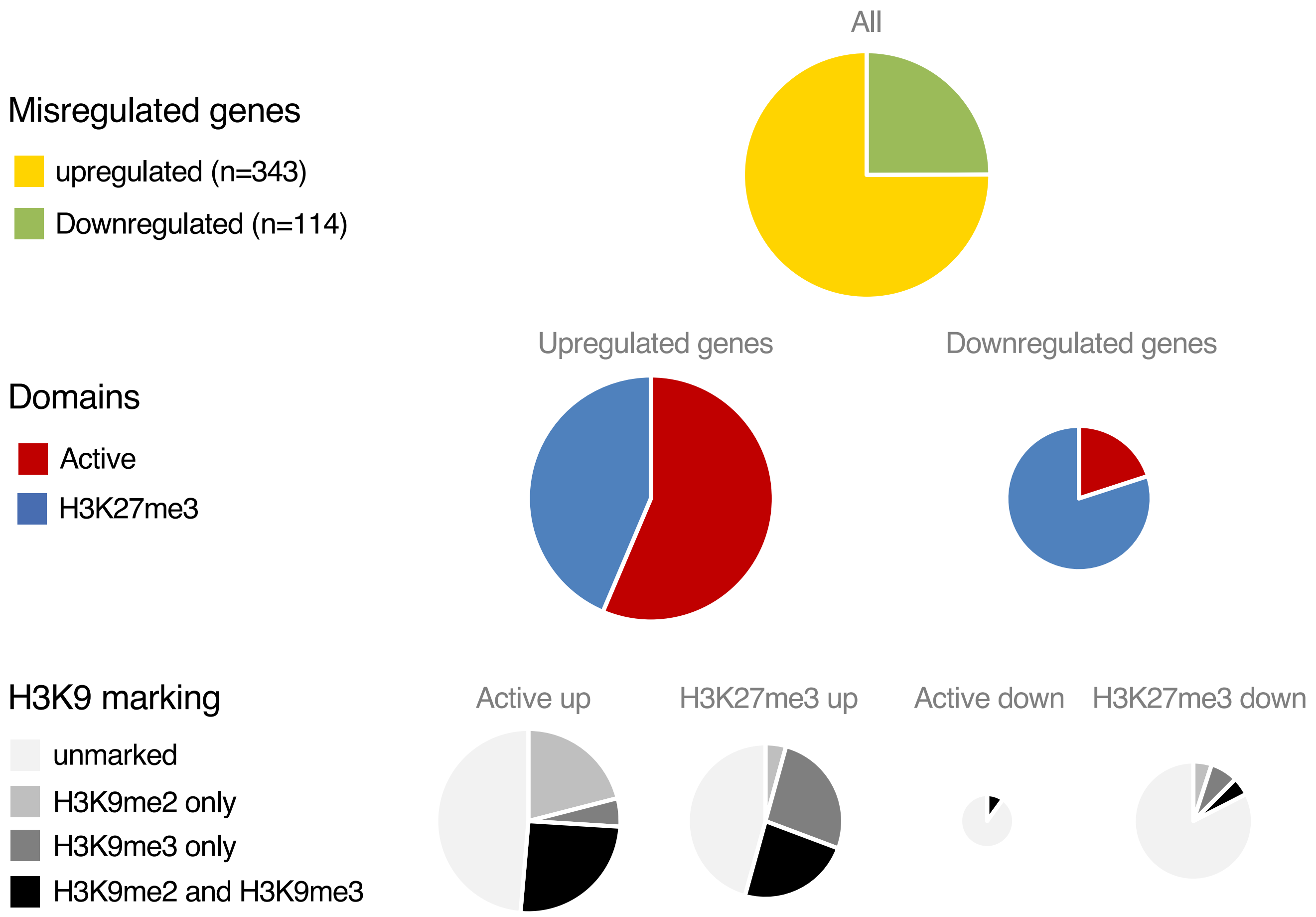

Supplemental Fig. S9 Relationship between gene expression changes in *met-2 set-25* mutants, domain type, and H3K9 methylation

Supplemental Figure S10

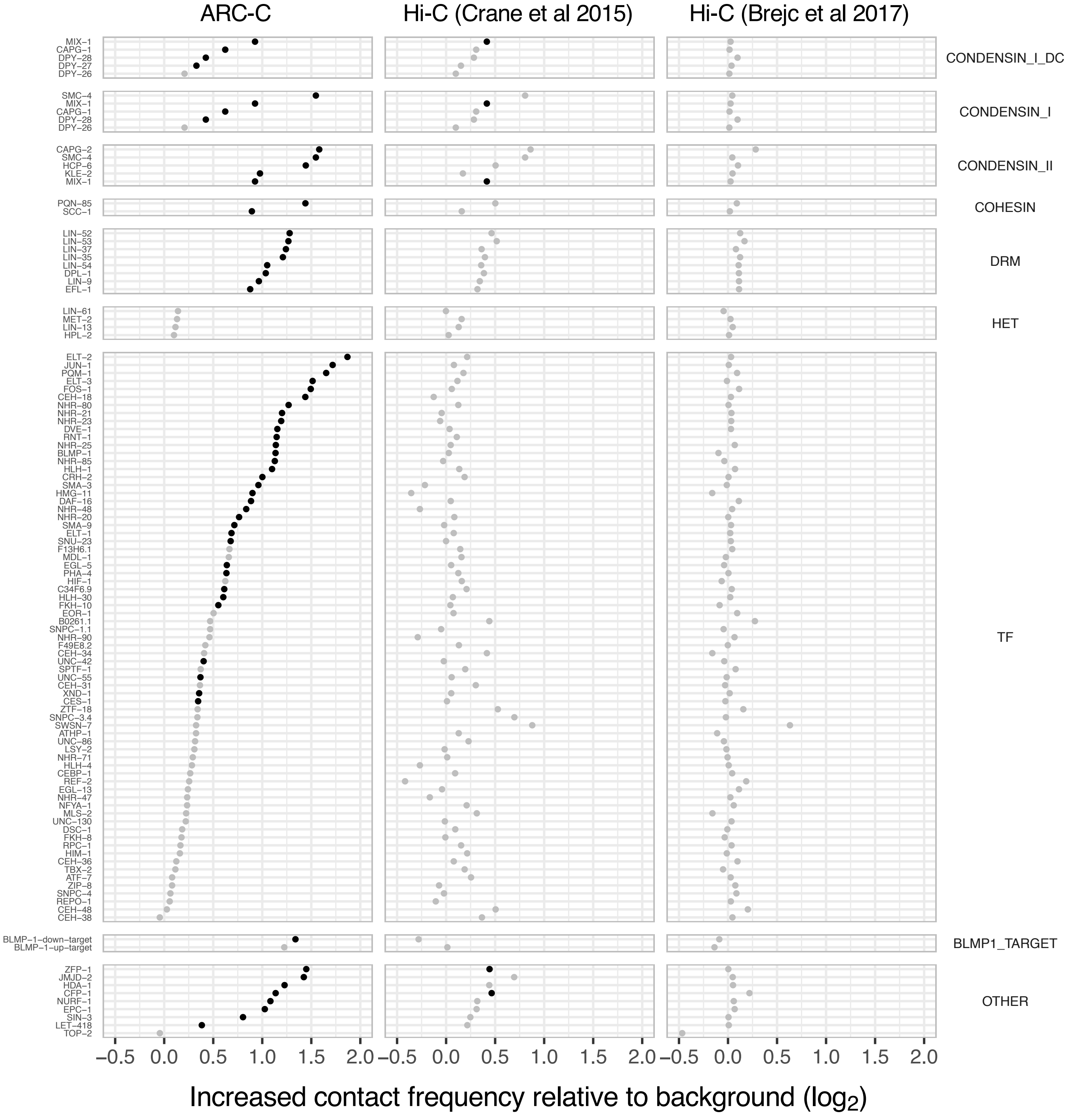

Supplemental Fig. S10 Comparison of contact frequency between binding sites of indicated proteins in ARC-C and Hi-C maps.

Black circles indicate significant enrichment. Hi-C data are from Crane et al. 2015 and Brejc et al 2017. See Methods for details.

Supplemental Figure S11

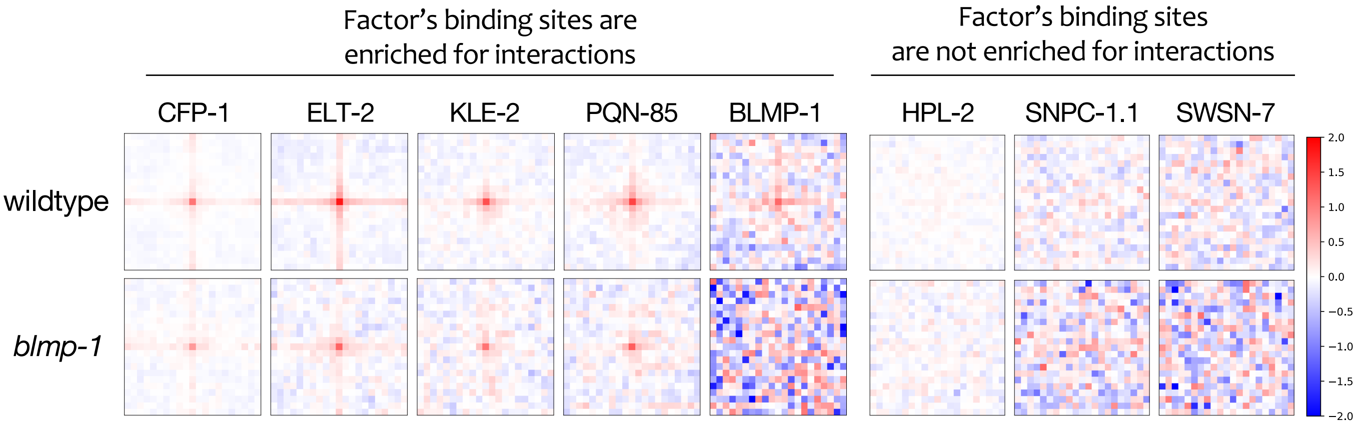

Supplemental Fig. S11 Aggregate contact analyses of transcription and chromatin factor binding regions

Representative aggregate contact analysis (APA) plots of data in Supplemental Fig. S8 showing ARC-C signal between binding sites of indicated factors at 1kb resolution and a distance range of 20kb-1Mb.

Supplemental Figure S12

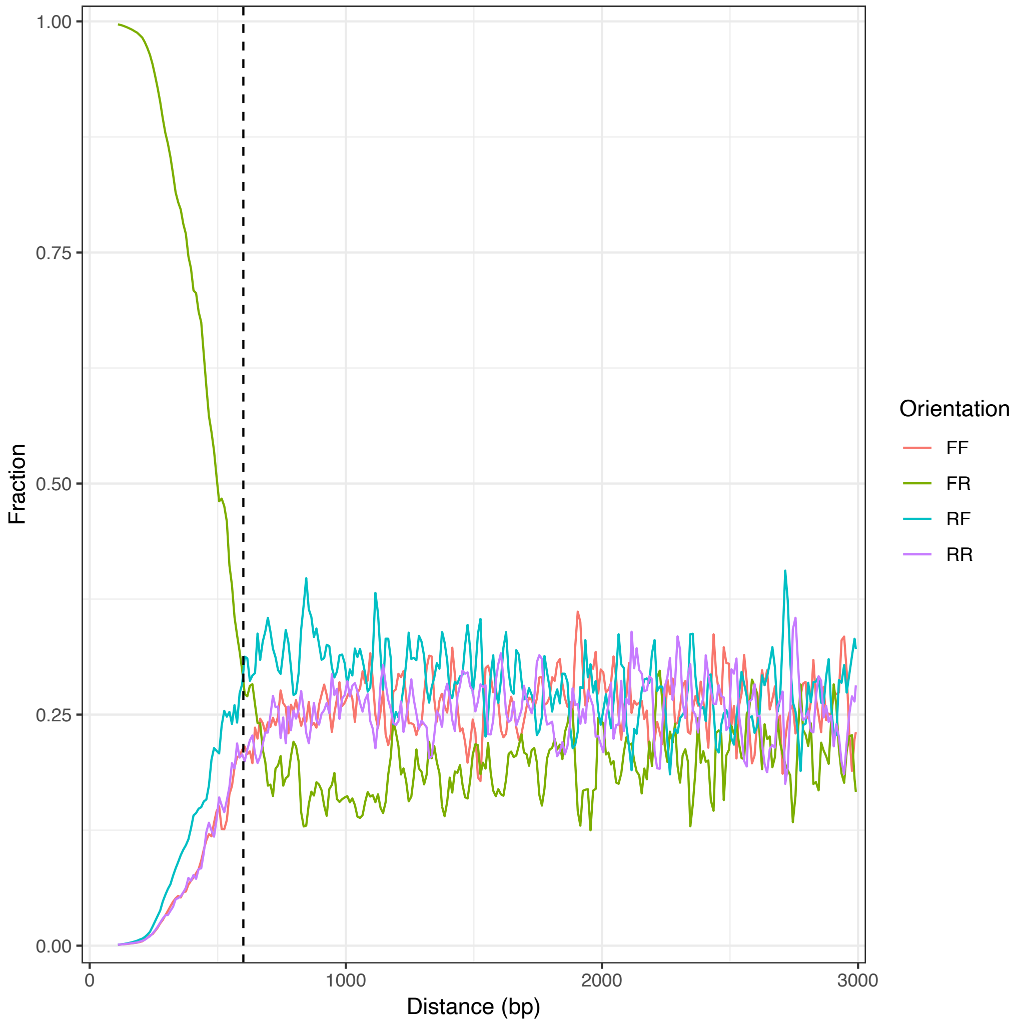

Supplemental Fig. S12 Fraction of read pair orientation as a function of mapping distance.

FF, both reads map in forward orientation; FR, read 1 maps in forward orientation and read 2 maps in reverse orientation; RF, read 1 maps in reverse orientation and read 2 maps in forward orientation; RR, both reads map in reverse orientation. Only read pairs where read1 mapped to a smaller coordinate than read2 are plotted. Reads are from the wild-type ARC-C map. Dashed vertical line marks 600bp.

Supplemental Figure S13

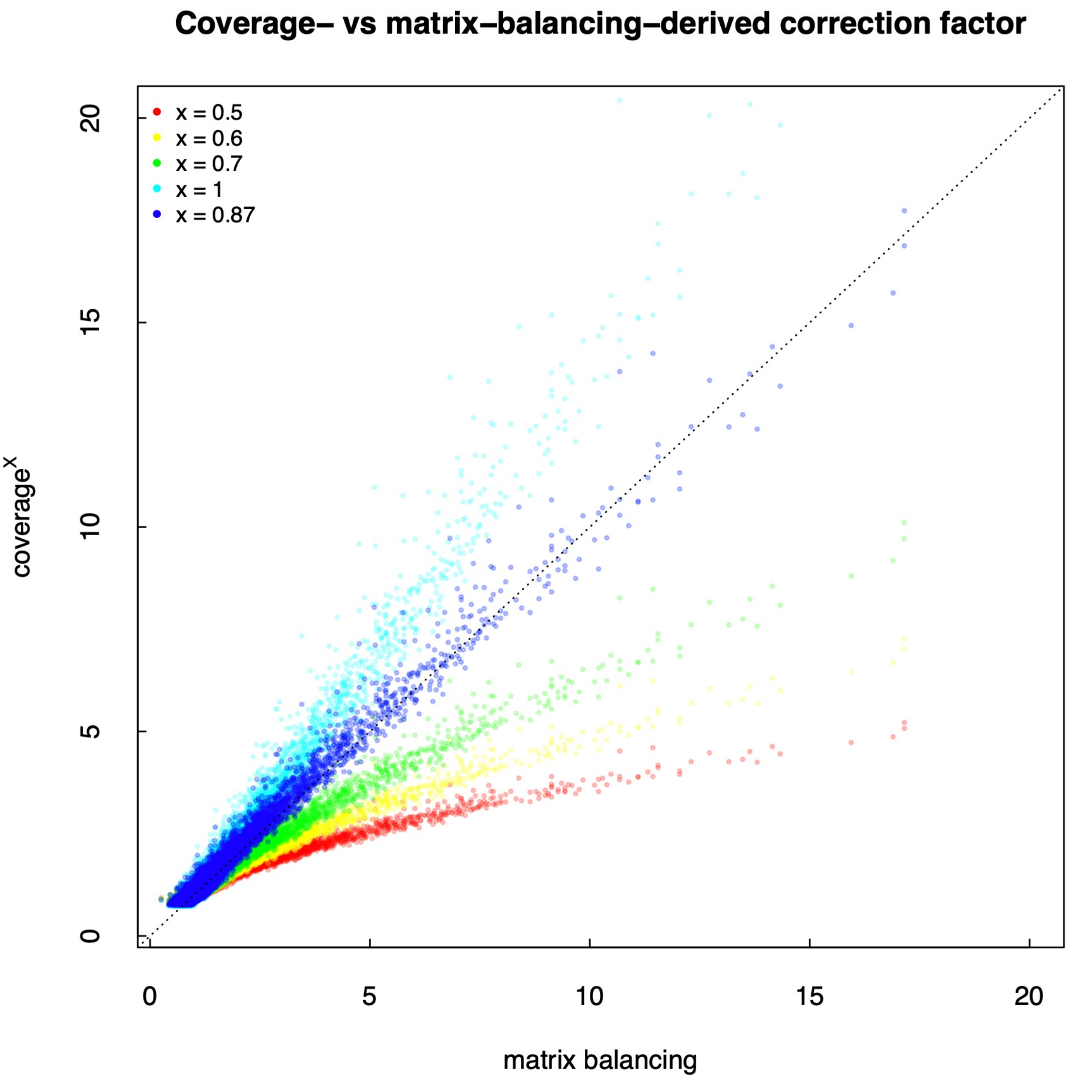

Supplemental Fig. S13 Comparing matrix-balancing-derived correction factors and reciprocal of different exponents of bin coverage

In the plot, the x axis gives matrix-balancing derived correction factors (normalised so that the median is 1), whereas y axis gives the reciprocal of different exponents of bin coverage (normalised so that the median is 1).
